# Delayed Diagnosis and Treatment of Cancer Patients During the COVID-19 Pandemic in Henan, China: An Interrupted Time Series Analysis

**DOI:** 10.3389/fpubh.2022.881718

**Published:** 2022-05-24

**Authors:** Changpeng Liu, Heng Piao, Tao Zhang, Dongjian Yang, Xiaoyan Li, Xiance Tang

**Affiliations:** ^1^Department of Medical Records, Office for DRGs (Diagnosis Related Groups), The Affiliated Cancer Hospital of Zhengzhou University, Henan Cancer Hospital, Zhengzhou, China; ^2^Department of Epidemiology, School of Public Health, Fudan University, Shanghai, China; ^3^Center for Medical Big Data, International Peace Maternity and Child Health Hospital, School of Medicine, Shanghai Jiao Tong University, Shanghai, China

**Keywords:** COVID-19, cancer, ARIMA, interrupted time series, Henan

## Abstract

**Objective:**

To investigate the possible impact of lockdown policies on the diagnosis and treatment of cancer patients in Henan, China.

**Design, Setting, and Participants:**

We collected data from the Henan Cancer Hospital, affiliated with Zhengzhou University. The monthly numbers of inpatient admissions from January 2014 to December 2019 were used to forecast the number of inpatient admissions in 2020, which was then compared to the actual number of patients admitted during the pandemic to evaluate how the actual number diverges from this forecast. We conducted an interrupted time series analysis using the autoregressive integrated moving average (ARIMA) model.

**Main Outcomes and Measures:**

For specific diagnoses, treatment modalities, and age groups, we compared the changes in monthly admissions after the pandemic with the forecasted changes from the model.

**Results:**

The observed overall monthly number of inpatient admissions decreased by 20.2% [95% confidence interval (CI), 11.7–27.2%], 78.9% (95% CI, 77.3–80.4%), and 40.9% (95% CI, 35.6–45.5%) in January, February, and March 2020, respectively, as compared with those predicted using the ARIMA model. After the lockdown, visits for all treatment modalities decreased sharply. However, apparent compensation and recovery of the backlog appeared in later surgeries. As a result, the number of patients who underwent surgery in 2020 (30,478) was close to the number forecasted by the ARIMA model (30,185). In the same period, patients who received other treatments or underwent examinations were 106,074 and 36,968, respectively; the respective numbers that were forecasted by ARIMA were 127,775 and 60,025, respectively. These findings depict a decrease of 16.9 and 38.4% in patients who received other treatments or underwent examinations only, respectively. Regarding diagnosis, the reported incidence of various cancers decreased dramatically in February, with varying extent and speed of recovery.

**Conclusion and Relevance:**

The COVID-19 pandemic has significantly delayed the diagnosis and treatment of cancer in Henan, China. Long-term research should be conducted to assess the future effects of lockdown policies.

## Introduction

The coronavirus 2019 (COVID-19) pandemic has disrupted the global health systems. As the number of patients with COVID-19 increased rapidly in 2020, more resources were channeled into health systems to manage the virus, leaving limited resources to be allocated for other diseases (1–4). Concurrently, governments launched lockdowns to restrict the movement and activities of citizens and to reduce community transmission. Such policies had a profound influence on various aspects of society, including employment, education, economy, and health (5, 6).

The prevention and control policies for controlling the spread of the virus taken by hospitals have played a positive role but have also impacted the treatment of other diseases. Cancer patients are of particular concern because they especially need timely treatment and diagnosis (7). Mounting evidence shows that the diagnosis and treatment of cancer were delayed during the pandemic (8), which may increase the likelihood of cancer metastasis or lead to progression from a curable to an incurable state (9). Therefore, understanding the impact of the pandemic and lockdown policies on the admission of cancer patients would improve the care of patients during the pandemic. It can also help in future public health policymaking and interventions. Disruption of cancer treatment, delay in treatment plans, and prolonged intervals between visits have been previously documented in some areas (1, 10–12). For instance, a significant decline in radiotherapy sessions, endoscopic services, and surgeries was reported in the UK during and after the lockdown, which significantly impacted the survival of affected patients (8, 9, 13). Reports from Catalonia (Spain) have revealed a reduced reported cancer incidence (14). In addition, the chaos that resulted from COVID-19 has had a substantial impact on cancer diagnosis in the Netherlands (9). Some studies in China have also reported the impact of COVID-19 on cancer patients. In Shanghai, the number of outpatients decreased significantly, especially out-of-town and elderly patients, while the number of patients undergoing chemotherapy and surgery remained unchanged (15). In Guangdong, hospital capacity was reduced by 28.00% during the lockdown (16). In Beijing, the number of patients scheduled for elective surgery for colorectal cancer decreased substantially, but the severity of their disease remained the same (17). However, studies from low- and middle-income areas in China are few, and such areas are underrepresented. Specifically, these studies are limited by small sample size and relatively low methodological quality. In addition, these studies compared patients admitted during the pandemic period to patients admitted 1 year before without taking autocorrelation, underlying long-term trends, and seasonality into consideration.

To take control of COVID-19, the Henan Provincial Government responded swiftly by implementing national lockdown policies and introducing additional measures on January 25, 2020, as follows (18). First, the government used television broadcasts to raise public awareness of the seriousness of COVID-19. Second, supervision was strengthened, and cities in Henan were ordered to conduct home quarantine monitoring for at least 14 days and to suspend all provincial transport operations. Third, comprehensive epidemic measures were taken. The Spring Festival holiday was canceled at all levels in the province. All medical workers were on standby 24 h a day and reported their locations to respond to any possible COVID-19 cases. Cities in Henan province took epidemic prevention and control measures at their own discretion. For instance, Zhengzhou halted all tourist services from outside the city and cut back on their public transport department. Nanyang delayed the start of government services, resumption of enterprises, opening of schools, and start of administrative approval services.

Here, we explored the influence of the COVID-19 pandemic and lockdown policies on cancer patients in Henan by interrupted time series (ITS) analysis using autoregressive integrated moving average (ARIMA) models, which take the autocorrelation, underlying long-term trends, and seasonality into consideration. As the only tertiary cancer hospital in Henan, Henan Cancer Hospital has a bed capacity of over 3,000, about 660,000 outpatient visits, and 180,000 hospitalizations annually (19). Henan Cancer Hospital is an oncology specialist hospital and, therefore, does not admit patients with COVID-19. However, the new policies hindered our clinical environment with relation to the treatment of new patients and the ongoing treatment and monitoring of postoperative patients in the outpatient clinic. These measures did not stop until the local infection rates dropped significantly in April 2021. Our clinical environment has gradually returned to normal following the reduction in local infection rates. We hypothesized that the reduction in the admissions of cancer patients was associated with lockdown measures. To test this hypothesis, we conducted an ITS analysis by following patients with cancer using a database from Henan Cancer Hospital.

## Materials and Methods

### Design and Data Source

An uncontrolled before-and-after study was conducted using electronic medical record data from the Henan Cancer Hospital, which is affiliated with Zhengzhou University and is the only tertiary tumor hospital in Henan Province. Ninety-eight percent of patients visiting Henan Cancer Hospital are from Henan, and they represent ~20% of all cancer patients in Henan. In 2016, hospitals in Henan admitted 670,335 inpatients diagnosed with cancer, with 121,947 of these being admitted to Henan Cancer Hospital. Demographic information of patients was exported from the Hospital Information System. Trained case managers coded admission and discharge diagnoses according to the International Classification of Diseases (ICD)-10.

The study was approved by the Medical Ethics Committee of Henan Cancer Hospital (no. 2021-KY-0054-001). The data were anonymized, and the requirement for informed consent was therefore waived.

### Participants and Study Period

All patients diagnosed with malignant neoplasms according to ICD-10 codes from January 2014 to December 2020 were included in the study. We divided the overall study period into three subperiods to evaluate the reductions in cancer admissions: “training period” from 2014 to 2019, “lockdown period” from January to March 2020, coinciding with the beginning of the lockdown, and “post lockdown period” from April to December 2020, which represents the period after the ease of lockdown.

### Main Outcomes

The number of malignant neoplasm diagnoses was the main variable. We also calculated the number of monthly admissions for malignant neoplasms.

### Statistical Analyses

We used both ARIMA and linear models, with each method having distinct benefits. The ARIMA is a ”true” time-series method that gives more accurate predictions, but its parameters are difficult to interpret, whereas the linear model can detect both trend and step changes. Time-series analyses were conducted by sex, age group (0–54, 55–69, ≥70 years old), treatment modality (surgery, non-surgical treatment, or examination only), and diagnosis (Supplementary Table 1).

#### ARIMA Model

The ARIMA model is a useful tool for assessing the impact of large-scale interventions. It accounts for autocorrelation, underlying long-term trends, and seasonality (20). The model constructed herein was written as (p, d, q) (P, D, Q)s (21).

where p: autoregressive model order

d: ordinary differences

q: the moving average model order

P: seasonal, autoregressive model order

D: the number of seasonal differences

Q: the seasonal moving-average model order

s: the length of a periodic pattern (in this study, s = 12).

The formula of the ARIMA model was expressed as:


$$\begin{array}{l} Y_{t}\;=\;\frac{\theta_{q}\left(B\right)\Theta_{Q}\left(B^{s}\right)at}{\Phi_{P}\left(B^{s}\right)\phi \left(B\right){\left(1-B\right)}^{d}{\left(1-B^{s}\right)}^{D}}\; \end{array}$$

where θ_*q*_(*B*): the operator of the moving-average model

$\Theta_{Q}\left(B^{s}\right)$: the operator of the seasonal moving-average model

ϕ(*B*): the operator of the autoregressive model

$\Phi_{P}\left(B^{s}\right)$: the operator of the seasonal autoregressive model

(1−*B*)^*d*^: the component of the ordinary differences

(1−*B*^*s*^)*D*: the component of the seasonal differences

*at*: white noise

Yt: the predicted variable (22).

The monthly number of admissions was used to construct the ARIMA model. First, to help induce stationarity, we determined that a first difference (d) was needed owing to the visible increasing trend before the subsidy change and that a seasonal difference (D) was needed owing to the seasonality of the series. Second, the autocorrelation function and partial autocorrelation function plots were plotted for the stationary series to establish alternative ARIMA models. We specified values of d = 1 (to induce stationarity) and D = 1 (owing to the presence of seasonality) and iteratively searched a series of potential ARIMA models for the one with the lowest Bayesian information criterion (BIC). We set the maximum values of p and q to 5 and those of P and Q to 2. Third, by referring to the two criteria, the optimal ARIMA model was determined: (a) the BIC and (b) Ljung-Box tests proved that the residual sequence of the model was white noise. Finally, the model with the lowest BIC was (1) × (0,1,0)_12_. The model was selected to forecast admissions in 2020 in the absence of the lockdown policy and to evaluate how the observed trend diverges from this forecast (20).

#### ITS Model

ITS with segmented linear regression analysis was used. We determined the potential effects of the lockdown policy on admission trends. A linear regression model for the pre-and post-implementation segments was specified to allow for the calculation of the monthly admissions trend before the intervention, the change in the level, and the post-intervention trend. Three time periods were considered: before the lockdown announcement (from January 2014 to December 2019), during the lockdown (from January 2020 to March 2019), and after the lockdown (from April 2020 to December 2020).

We applied linear regression to determine the long-term trend and step change at the start of the lockdown as follows:


$$\begin{array}{l} Y_{t}\;=\;\beta_{0}\;+\;\beta_{1}T\;+\;\beta_{2}L\;+\;\beta_{3}X_{t}\;+\;\beta_{4}TX_{t}\;+\;\beta_{5}Z\; \end{array}$$

Where *Y*_*t*_ denotes the monthly number of cancer patients; β_0_ represents the regression intercept (monthly number of patients before lockdown); β_1_ denotes the coefficient of the slope before lockdown (long-term trend before lockdown); β_2_ denotes the difference in the second period from the pre-lockdown period; β_3_ represents the immediate step-change in the number of admissions in the third period; β_4_ denotes the change in trend in the third period; β_1_+β_4_ represents the time trend after the third period (coefficient of the slope); β_5_ denotes the coefficient of the Spring Festival; *T* represents the number of months from January 2014; *L* denotes the binary dummy variable indicating the second stage of lockdown; *X*_*t*_ represents the post-third periods coded as a binary variable; *TX*_*t*_ represents the time–lockdown interaction term in the third period; and *Z* represents a dummy variable representing the Spring Festival.

All analyses were performed using SAS software (version 9.4; SAS Institute, Cary, NC, USA) and R software (version: 3.6.2), with a *p* < 0.05 indicating statistical significance, and a 95% confidence interval (CI).

## Results

### Characteristics of the Study Subjects

There were 985,274 admissions to Henan Cancer Hospital from the 1^st^ of January 2014 to the 31^st^ of December 2020 (Table 1). Of all patients, 49.6% were aged 0–54 years (*n* = 488,421), and more females were admitted than males (54.2 vs. 45.8%, respectively). The most common reason for visiting the hospital was to receive chemotherapy (46.7%, *n* = 421,091). We found a significant difference in the frequency of surgical procedures between patients diagnosed with different cancer types, with the highest being in patients with thyroid cancer (34.2%, *n* = 8,257) and the lowest in patients with breast cancer (9.6%, *n* = 15,951). The most common reasons for admission were ranked as follows: breast cancer (16.8%, *n* = 165,882), lung cancer (15.2%, *n* = 149,435), and stomach cancer (8.8%, *n* = 866,332). The median number of monthly admissions was 11,729, and the mean was 13,909 [standard deviation (SD), 3011.28]. The number of admissions increased from 99,501 in 2014 to 183,347 in 2019 but decreased to 161,449 in 2020.

**Table 1:** Characteristics of inpatients from 2014 to 2020 in Henan Cancer Hospital.

	**All**	**Breast**	**Lung**	**Stomach**	**Lymphoid^a^**	**Gynecologic^b^**	**Esophagus**	**Rectum**	**Liver^c^**	**Colon**	**Thyroid**
**Age-group (years)**											
0–54	48,8421 (49.6)	11,6374 (70.2)	47,300 (31.7)	26,917 (31.1)	49,040 (60.9)	44,197 (58.9)	8,804 (16.2)	20,952 (41.7)	19,174 (45.3)	17,952 (45.1)	18,130 (75.1)
55–69	394,424 (40)	46,004 (27.7)	80,605 (53.9)	46,240 (53.4)	24,784 (30.8)	26,883 (35.9)	32,947 (60.5)	23,211 (46.2)	19,525 (46.1)	17,141 (43)	5,180 (21.5)
70-	102,429 (10.4)	3,504 (2.1)	21,530 (14.4)	13,475 (15.6)	6,659 (8.3)	3,917 (5.2)	12,729 (23.4)	6,065 (12.1)	3,673 (8.7)	4,736 (11.9)	835 (3.5)
**Sex**											
Male	445,167 (45.2)	739 (0.5)	95,233 (63.7)	64,392 (74.3)	45,449 (56.5)	0	38,269 (70.2)	29,098 (57.9)	34,395 (81.2)	23,041 (57.9)	6,629 (27.5)
Female	540,107 (54.8)	165,143 (99.6)	54,202 (36.3)	22,240 (25.7)	35,034 (43.5)	74,950 (100)	16,211 (29.8)	21,130 (42.1)	7,977 (18.8)	16,788 (42.2)	17,516 (72.6)
**Treatment**											
Chemotherapy	421,091 (42.7)	101,898 (61.4)	68,632 (45.9)	49,474 (57.1)	39,233 (48.8)	39,546 (52.7)	21,396 (39.3)	27,746 (55.2)	5,995 (14.2)	23,675 (59.4)	237 (1)
Surgery	211,669 (21.5)	15,951 (9.6)	33,991 (22.8)	13,933 (16.1)	19,691 (24.5)	11,769 (15.7)	13,877 (25.5)	6,323 (12.6)	12,413 (29.3)	5,045 (12.7)	8,257 (34.2)
Test	162,342 (16.5)	20,823 (12.6)	5,811 (3.9)	10,859 (12.5)	3,264 (4.1)	9,792 (13.1)	5,889 (10.8)	7,557 (15.1)	12,384 (29.2)	4,608 (11.6)	14,666 (60.7)
Radiotherapy	34,680 (3.5)	8,368 (5)	6,654 (4.5)	641 (0.7)	880 (1.1)	5,936 (7.9)	4,200 (7.7)	1,467 (2.9)	287 (0.7)	155 (0.4)	298 (1.2)
Targeted	19,906 (2)	4,425 (2.7)	6,253 (4.2)	893 (1)	906 (1.1)	779 (1)	479 (0.9)	975 (1.9)	662 (1.6)	1,019 (2.6)	57 (0.2)
Immunotherapy	17,244 (1.8)	1,411 (0.9)	4,013 (2.7)	1,251 (1.4)	1,400 (1.7)	968 (1.3)	937 (1.7)	520 (1.0)	1,540 (3.6)	579 (1.5)	58 (0.2)
Other	11,8342 (12)	13,006 (7.8)	24,081 (16.1)	9,581 (11.1)	15,109 (18.8)	6,207 (8.3)	7,702 (14.1)	5,640 (11.2)	9,091 (21.5)	4,748 (11.9)	572 (2.4)
**Year**											
2014	99,501 (10.1)	17,376 (10.5)	13,406 (9)	8,270 (9.6)	6,866 (8.5)	7,910 (10.6)	5,653 (10.4)	4,573 (9.1)	4,540 (10.7)	3,183 (8)	2,762 (11.4)
2015	11,0554 (11.2)	19,442 (11.7)	15,026 (10.1)	9,836 (11.4)	8,125 (10.1)	8,552 (11.4)	6,610 (12.1)	5,414 (10.8)	4,610 (10.9)	3,940 (9.9)	3,206 (13.3)
2016	12,0882 (12.3)	20,643 (12.4)	16,840 (11.3)	10,806 (12.5)	8,954 (11.1)	9,914 (13.2)	7,528 (13.8)	6,457 (12.9)	5,174 (12.2)	4,788 (12)	3,153 (13.1)
2017	14,2964 (14.5)	23,548 (14.2)	21,168 (14.2)	12,951 (15)	11,706 (14.5)	11,522 (15.4)	8,450 (15.5)	7,536 (15)	5,969 (14.1)	5,894 (14.8)	3,194 (13.2)
2018	166,577 (16.9)	30,467 (18.4)	25,596 (17.1)	14,584 (16.8)	14,312 (17.8)	12,488 (16.7)	9,426 (17.3)	8,342 (16.6)	6,839 (16.1)	6,783 (17)	3,637 (15.1)
2019	183,347 (18.6)	29,081 (17.5)	30,293 (20.3)	17,193 (19.9)	15,945 (19.8)	12,703 (16.9)	9,502 (17.4)	9,836 (19.6)	7,697 (18.2)	8,254 (20.7)	4,373 (18.1)
2020	161,449 (16.4)	25,325 (15.3)	27,106 (18.1)	12,992 (15)	14,575 (18.1)	11,908 (15.9)	7,311 (13.4)	8,070 (16.1)	7,543 (17.8)	6,987 (17.5)	3,820 (15.8)

a *Lymphoid, hematopoietic, and related tissue cancers*;

b *female gynecologic cancers*;

c *liver and intrahepatic bile ducts cancers*.

### Change in the Overall Number of Inpatients

**Figure 1:**
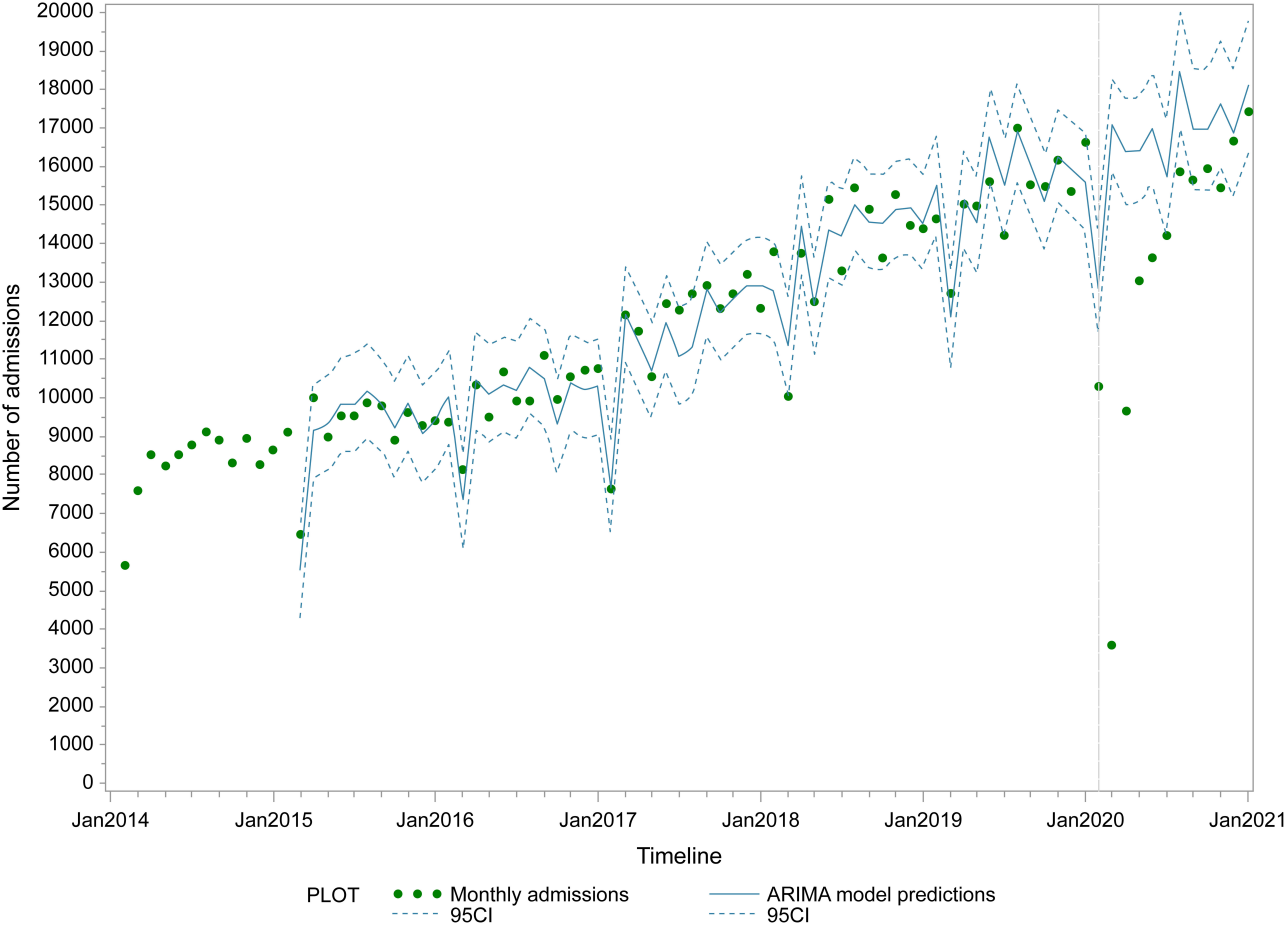
Overall monthly admissions before and after the COVID-19 lockdown in Henan Cancer Hospital. Points represent monthly number of hospital admissions. Vertical lines represent change-point (lockdown). Fitted line represents ARIMA regression model of the admissions. Surrounding dashed lines represent 95% confidence intervals.

Table 2 outlines the reported estimated coefficients of the linear model, while the ARIMA model predictions and the predicted number of admissions under the counterfactual scenario are plotted in Figure 1. We reported a linear increase in the number of admissions until January 2020, followed by a sharp reduction (Figure 1, Supplementary Table 2). Before the lockdown in January 2020, the number of admissions increased with a trend of 120.8 per month (95% CI: 109.4–132.1) from 5,662 in January 2014 to 16,621 in December 2019. In the first 3 months of lockdown, segmented regression analysis demonstrated a mean decrease of 7,636.4 (95% CI: 6,368.3–8,904.6). The decrease in admissions was already substantial by February 2020, with more than 70% of the number under the counterfactual scenario. The overall monthly admissions count was 10,289 in January, 3,597 in February, and 9,662 in March. In comparison to the ARIMA model predictions, the overall monthly number of inpatients decreased by 20.2% (95% CI: 11.7–27.2%), 78.9% (95% CI: 77.3–80.4%), and 40.9% (95% CI: 35.6–45.5%) in January, February, and March 2020, respectively. After April 2020, admissions increased by 485.0 (95% CI: 216.4–753.4) per month, reaching 90.7% of counterfactual levels (95% CI: 82.9–100.1%) by July 2020.

**Table 2:** Results of linear regression for the interrupted time series analysis.

**Variable**	**Estimate**	**95% CI**	***t***	***P*-value**
β0	7195.8	6712.3 to 7679.4	29.2	<0.0001
β1	120.8	109.4 to 132.1	20.9	<0.0001
β2	−7636.4	−8904.6 to −6368.3	−11.8	<0.0001
β3	−3355.3	−4883.9 to −1826.7	−4.3	<0.0001
β4	364.2	107 to 621.3	2.8	0.002
β5	−1939.1	−2742.5 to −1135.7	−4.7	<0.0001

### Stratified Analysis by Sex, Age, and Treatment Modality

**Figure 2:**
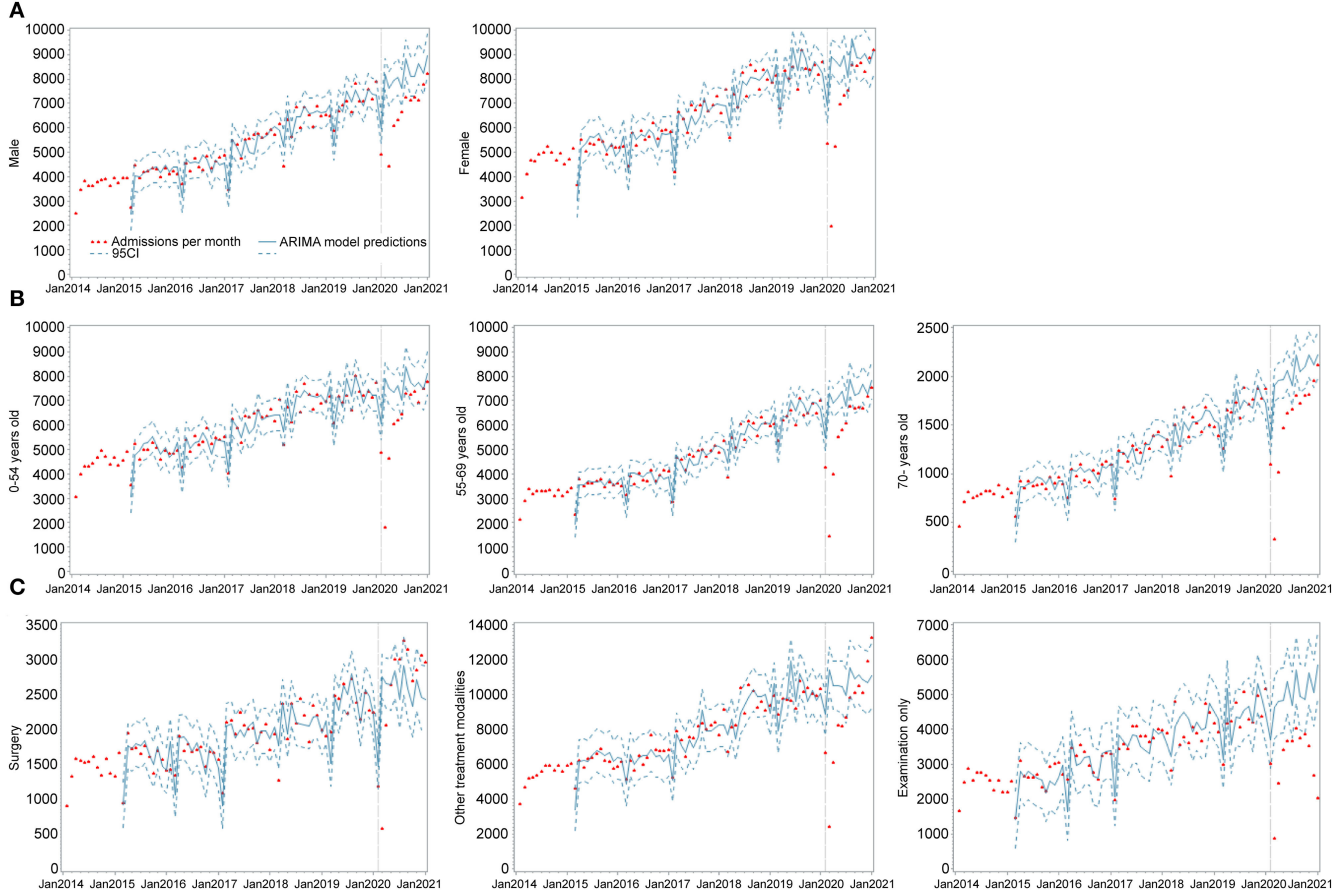
Monthly admissions before and after the COVID-19 lockdown in Henan Cancer Hospital stratified by sex, age group, and treatment modalities. **(A)** Admissions by sex. **(B)** Admissions by age group. **(C)** Admissions by treatment modalities. Vertical lines represent change-point (lockdown). Fitted lines represent ARIMA regression models of the admissions. Surrounding dashed lines represent 95% confidence intervals.

Figure 2 shows the results of the interrupted time-series analyses stratified by sex, age, and treatment modality (Supplementary Table 2). Trends in admissions across sexes displayed similar patterns to the overall trends, albeit with some variations (Supplementary Table 2). According to the plots of the fitted model, male admissions were slower to recover than those of females. In terms of age, we found subtle variations in trends between the different groups. Substantial variation was reported across different treatment modalities in both the direction and magnitude of change in monthly admissions when compared to the number predicted by the ARIMA model.

Visits for all kinds of reasons decreased in January and gradually returned to their previous levels, although the speed and extent of this recovery varied. The number of patients presenting for surgery and other treatments appeared to recover more quickly than that of patients presenting for examination only. Furthermore, the number of patients whose reason for visiting was examination continued to drop even after June 2020 (Figure 2). The number of patients admitted for surgery recovered to match the predicted level by March 2020 with temporary level change, which is much faster than other treatment options. Moreover, the number of patients who underwent surgery surpassed the number predicted by the ARIMA model by July 2020. A total of 30,478 patients underwent surgery in 2020, nearly the same as the number forecasted by the ARIMA model (30,185, 95% CI: 25,350–35,017). By comparison, the number of patients who received other treatment modalities increased with a slope change, surpassing the number predicted by the ARIMA model by November 2020. During the same period, the number of patients who received other treatment modalities and that of patients who underwent examinations only were 106,074 and 36,968, respectively, while the numbers forecasted by the ARIMA model were 127,775 (95% CI: 109,058–146,485) and 60,025 (95% CI: 48,873–71,175), representing a decrease of 16.9 and 38.4%, respectively.

### Stratified Analysis by Diagnosis

**Figure 3:**
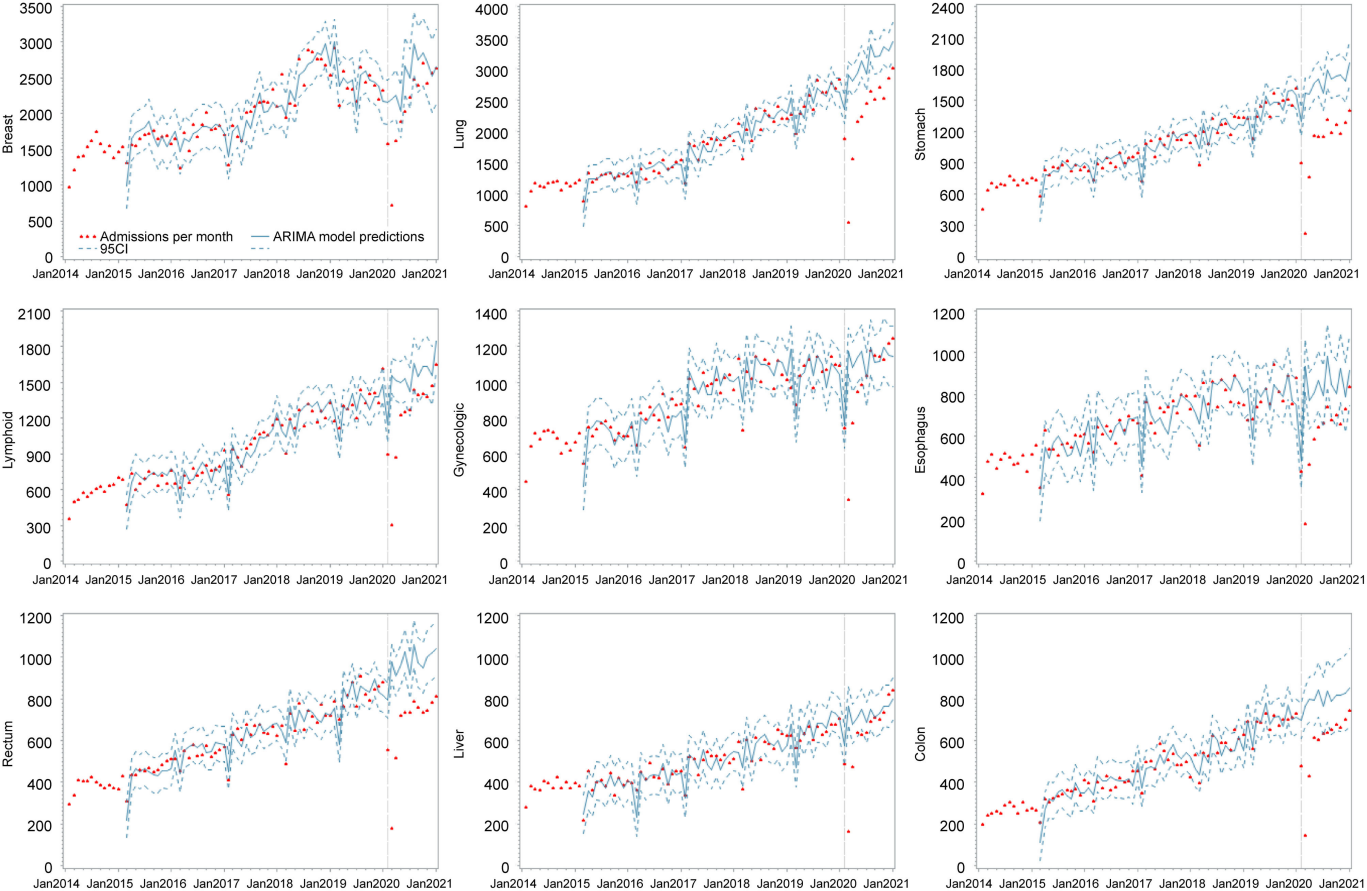
Monthly admissions before and after the COVID-19 lockdown in Henan Cancer Hospital according to diagnosis. Breast: breast cancer; Lung: lung cancer; Stomach: stomach cancer; Lymphoid: lymphoid, hematopoietic, and related tissue cancers; gynecologic: female gynecologic cancers; Esophagus: esophageal cancer; Rectum: rectal cancer; Liver: liver and intrahepatic bile ducts cancers; Colon: colon cancer. Vertical lines represent change-point (lockdown). Fitted lines represent ARIMA regression models of the admissions. Surrounding dashed lines represent 95% confidence intervals.

Changes in monthly admissions stratified by diagnosis are shown in Figure 3. We observed the largest reduction in admissions in 2020 among patients with lung cancer [with a decrease of 9,813 (−26.6%) from 36,919] and stomach cancer [a decrease of 6,899 (−34.7%) from 19,891 for the same period]. A dramatic increase in the detection of various cancers was reported in February, although the extent and speed of this recovery varied. By December 2020, the number of patients diagnosed with lung, stomach, lymphoid, esophageal, rectal, and colon cancer did not recover to the counterfactual level. Conversely, more patients were admitted for cancer in December 2020 compared to the equivalent month forecasted by the ARIMA model. In fact, the number of female gynecologic cancer cases increased by 101 (8.8%) from 1,147, and the number of colon cancer cases increased by 105 (12.6%) from 799.

## Discussion

The number of admissions in Henan fell during the implementation of the lockdown policy. In particular, more significant reductions of 78.9, 40.9, and 20.6% were reported in February, March, and April, respectively. In the first 3 months after the lockdown, we found a reduction of 23,554 inpatients, representing 47% fewer cancers than expected. The number of admissions did not fully recover to its previous level by the end of 2020, especially for individuals with specific cancer types.

Our findings are in accordance with previous results (8, 10, 23–27). For example, the number of urgent referrals in the UK decreased by 34.3% between February and April 2020 (12), with a reduction of 70–89% in the number of cancer diagnoses for the same period as reported by Williams et al. (23). A decrease in the diagnosis of cancers in the Netherlands reached 25% for all cancers (excluding skin cancer) and 60% for skin cancers. Moreover, a study in Slovenia revealed that X-ray scans, mammograms, and ultrasounds decreased by 48, 75, and 42%, respectively (10). There are several explanations for this phenomenon. First, patients with non-specific symptoms of cancer may not consult a general practitioner, for example, because of being afraid of COVID-19 transmission in a hospital setting. Second, there were several barriers to visiting clinics during the lockdown, such as the halting of public transportation and the requirement of providing a justified reason for commuting (28). Third, hospitals were more likely to postpone admissions during the pandemic. This is because many medical resources were channeled to COVID-19 management, and the physical distancing policy barred hospitals from admitting more patients. Besides the lockdown policy, Henan Cancer Hospital had taken infection control measures to mitigate transmission of COVID-19. For example, the temperature of patients was screened at the hospital entrance. Patients with symptoms, such as cough, shortness of breath, or fever, were isolated and tested immediately. A physical distancing policy was implemented in the waiting area. Visitors from outside the hospital were banned. However, there were few changes in the care of cancer patients during the lockdown.

In addition to the overall changes in the number of admissions, we explored how these changes varied by treatment modality. There was a dramatic decrease in the number of surgeries at the beginning of the pandemic. However, it swiftly normalized to its previous levels, and most patients who missed a surgery underwent the operation at a later time, implying that the overall number of surgical procedures remained unchanged during 2020. However, for cancer patients, delayed surgery potentially increases the risk of tumor metastasis, and some cancers may progress from curable to non-curable during this period of delay (9, 29). There is evidence that the number of patients receiving radiotherapy, chemotherapy, and other non-surgical treatments in Henan Cancer Hospital dropped significantly during the implementation of the lockdown policy, with recovery after the first 3 months of 2020. A 17.0% reduction in these non-surgical treatment procedures was seen in 2020 compared to the counterfactual scenario. However, the number of patients who underwent examinations only declined further after June 2020. In the first few months of the lockdown, when surgery and other treatments were reduced by 80%, it was necessary to allocate extraordinary resources to deal with the backlog of cases. Some studies revealed that the decrease in diagnosis of cancer patients could also be linked to a reduction in medical encounters, consequently reducing diagnostic suspicion (10) and, therefore, delayed treatment (30).

The present study demonstrated that the lockdown policy influenced patients with nearly all cancer types. The decrease in diagnosis rates during the first month of lockdown ranged between 67.2% for breast cancer and 86.0% for stomach cancer, which is different from the impact reported in several other studies. Italy had the largest decrease in the diagnosis of patients with colorectal cancer and prostate cancer, accounting for 62 and 75%, respectively, while patients with breast cancer were the least affected, with a reduction of 26% (11). In the UK, the number of diagnosed melanoma cases decreased by 67.1%, but that of lung cancer cases reduced moderately (46.8%) (24). There was also a discrepancy in the decrease between non-skin and skin cancers (26 vs. 60%, respectively) in the Dutch population (27). Compared to other studies, the number of admissions in Henan fell more steeply, and the variance between cancer types was smaller. This may be because the Chinese government responded more quickly to what started as an epidemic, and citizens were more willing to comply with prevention and control policies for the epidemic (31–33). Mainland China effectively managed the spread of COVID-19 through a series of strict physical isolation policies, extensive early detection measures, and contact tracing strategies, which possibly reduced the duration of lockdown.

Furthermore, admissions of patients of different ages and diagnoses dropped almost equally sharply at the beginning of the lockdown in our study, but with varying speeds of recovery. Although the effect of the lockdown policy may be effective in halting the epidemic, delays in cancer diagnosis and treatment, which are associated with negative health outcomes, should be accounted for. When treating certain low-risk cancers, such as skin cancer, the effect of a moderate delay in treatment on survival and quality of life is minimal. In contrast, for lung cancer, pancreatic cancer, or breast cancer, which progress rapidly, immediate diagnosis and treatment are required to prevent adverse consequences. In the UK, patients over 70 years of age had a greater decline in the radiotherapy course than those under 70 years of age because in the high-risk former group, clinicians and patients decided to defer treatment. Additionally, the decrease occurred mainly in cancers for which treatment could be safely delayed (8). Therefore, it is imperative to assess patients of different ages and with various cancer types when making decisions regarding whom to prioritize during the lockdown period.

This study has some limitations. First, the ARIMA model is a “true” time-series model that provides more accurate predictions than the linear model, although its parameters are difficult to interpret, whereas the linear model can detect both trend and step changes. Moreover, it is subjective and challenging to choose the most appropriate ARIMA model. Hence, we also used a linear model to interpret the results. Second, although there were no other major events during the lockdown that might have affected the treatment of cancer patients, we cannot exclude changes in epidemiology, health care needs, weather, and sociocultural factors, which may have influenced the results. Third, we only analyzed the medical records of a single hospital. However, ~20% of cancer patients in Henan are treated in Henan Cancer Hospital. As Henan is a mid-income province, it is reasonable to believe that the pattern discovered in our study represents the overall scenario in China. Studies in other areas indeed revealed decrease in the numbers of cancer patients during the COVID-19 pandemic in China (15–17). Finally, our results demonstrated the diagnostic data only of inpatients and not those of outpatients. The rate of outpatients to inpatients was ~1:4 (according to hospital reports during the study period) and there was no shift from inpatients to outpatients during the pandemic. It is paramount that future research is conducted with a multicenter design and a larger number of enrolled hospitals, including as much diagnostic data of outpatients as possible.

In brief, the lockdown imposed because of COVID-19 has significantly delayed the diagnosis and treatment of cancer, which is a disease that carries high risks if not rapidly diagnosed and effectively managed. Only 1,273 confirmed COVID-19 cases were reported in Henan Province by June 17, 2020, and the cumulative incidence was 1.33 per 100,000 (18). In addition, Henan Cancer Hospital is an oncology specialist hospital and does not admit patients with COVID-19. The direct impact of COVID-19 circulation and overload of the healthcare system on cancer patients is minimal when compared with the lockdown policy. A series of factors jointly led to the reduction of access to hospitals, including mandatory movement restriction, fear of being infected, and physical distancing policies barring the hospital from admitting patients. Although strict lockdown policies effectively controlled the COVID-19 epidemic in China, they hindered the access to healthcare as well. Several studies have demonstrated an association between treatment delays and increased mortality. A study in the USA discovered a statistically significant association between delayed surgery and adverse outcomes in cancer patients (34). The number of avoidable cancer deaths in the UK is expected to rise considerably because of delays in diagnosis caused by COVID-19 (35). Urgent public health interventions are required to mitigate the expected impact of the pandemic on cancer patients. Pre-emptive public education is needed to encourage patients with cancer symptoms to see the doctor, and barriers to hospital access should be reduced. Telemedicine should be used as it not only helps patients who cannot visit the hospital in person but also reduces the backlog after the lockdown (18). As much as we must continue to manage the spread of COVID-19, there is a need to ensure that delayed diagnosis and treatment of cancer patients are accounted for when making decisions. Physicians should continue management aimed at treating high-risk tumors despite the viral prevalence, as mortality from untreated active malignancy is extremely high. Long-term research should be conducted to assess the future effects of lockdown policies.

## Data Availability Statement

The data that support the findings of this study are available on request from the corresponding author.

## Ethics Statement

The studies involving human participants were reviewed and approved by the Medical Ethics Committee of Henan Cancer Hospital. The Ethics Committee waived the requirement of written informed consent for participation.

## Author Contributions

CL, HP, and XT contributed substantially to the conceptualization and writing of this article. All authors contributed to writing of the article and approved the submitted version.

## Funding

The study was supported by grants RKX202002007 and LHGJ20200193 from the Key Science and Technology Program of Henan Province, China.

## Conflict of Interest

The authors declare that the research was conducted in the absence of any commercial or financial relationships that could be construed as a potential conflict of interest.

## Publisher's Note

All claims expressed in this article are solely those of the authors and do not necessarily represent those of their affiliated organizations, or those of the publisher, the editors and the reviewers. Any product that may be evaluated in this article, or claim that may be made by its manufacturer, is not guaranteed or endorsed by the publisher.
