# Corrigendum: Delayed diagnosis and treatment of cancer patients during the COVID-19 pandemic in Henan, China: An interrupted time series analysis

**DOI:** 10.3389/fpubh.2023.1164116

**Published:** 2023-02-28

**Authors:** Changpeng Liu, Heng Piao, Tao Zhang, Dongjian Yang, Xiaoyan Li, Xiance Tang

**Affiliations:** ^1^Department of Medical Records, Office for DRGs (Diagnosis Related Groups), The Affiliated Cancer Hospital of Zhengzhou University, Henan Cancer Hospital, Zhengzhou, China; ^2^Department of Epidemiology, School of Public Health, Fudan University, Shanghai, China; ^3^Center for Medical Big Data, International Peace Maternity and Child Health Hospital, School of Medicine, Shanghai Jiao Tong University, Shanghai, China

**Keywords:** COVID-19, cancer, ARIMA, interrupted time series, Henan

In the original article, **affiliation [1]** was incorrectly written “Department of Medical Records, Office for DRGs (Diagnosis Related Groups), Henan Cancer Hospital, Affiliated Cancer Hospital of Zhengzhou University, Zhengzhou, China.” The correct affiliation should be corrected as follows:

“Department of Medical Records, Office for DRGs (Diagnosis Related Groups), The Affiliated Cancer Hospital of Zhengzhou University, Henan Cancer Hospital, Zhengzhou, China”

The authors apologize for this error and state that this does not change the scientific conclusions of the article in any way. The original article has been updated.

