# Supplementary material for: Delayed Diagnosis and Treatment of Cancer Patients During the COVID-19 Pandemic in Henan, China: An Interrupted Time Series Analysis
Source: Front Public Health. 2022 May 24;10:881718. doi: 10.3389/fpubh.2022.881718 (PMC9171044; doi:10.3389/fpubh.2022.881718)
Supplement: Supplementary file 1 [file Table_1.DOCX]

Supplementary Material

## Supplementary Tables

**Supplementary Table 1.** Diagnostic groupings by ICD-10 code

| Diagnosis | ICD-10 code |
| --- | --- |
| Breast | C50, D05 |
| Lung | C33, C34 |
| Stomach | C16 |
| Esophagus | C15 |
| Rectum | C20 |
| Liver and intrahepatic bile ducts | C22 |
| Colon | C18 |
| Female gynecologic cancers | C51-C58 |
| Thyroid | C73 |
| Lymphoid, hematopoietic, and related tissues | C81-C96 |

**Supplementary Table 2.** Differences in observed and predicted admissions after the COVID-19 lockdown stratified by sex, age group, and treatment modality

|  | January | February | March | April | May | June | July | August | September | October | November | December |
| --- | --- | --- | --- | --- | --- | --- | --- | --- | --- | --- | --- | --- |
| All |  |  |  |  |  |  |  |  |  |  |  |  |
| Observed | 10289 | 3597 | 9662 | 13054 | 13642 | 14213 | 15862 | 15667 | 15930 | 15439 | 16657 | 17437 |
| Predicted | 12887 | 17077 | 16359 | 16431 | 17016 | 15677 | 18464 | 16969 | 16946 | 17625 | 16851 | 18108 |
| Change | -20.2 | -78.9 | -40.9 | -20.6 | -19.8 | -9.3 | -14.1 | -7.7 | -6 | -12.4 | -1.2 | -3.7 |
| Lower 95% CI | -11.7 | -77.3 | -35.6 | -13.2 | -12.4 | 0.1 | -6.4 | 1.6 | 3.7 | -3.5 | 9.7 | 6.3 |
| Upper 95% CI | -27.2 | -80.4 | -45.5 | -26.7 | -26.1 | -17.1 | -20.6 | -15.4 | -14.1 | -19.8 | -10 | -12 |
| Male |  |  |  |  |  |  |  |  |  |  |  |  |
| Observed | 4923 | 1633 | 4431 | 6088 | 6320 | 6654 | 7273 | 7126 | 7254 | 7126 | 7799 | 8227 |
| Predicted | 5988 | 8187 | 7617 | 7929 | 8086 | 7643 | 8835 | 8107 | 8115 | 8614 | 8234 | 8971 |
| Change | -17.8 | -80.1 | -41.8 | -23.2 | -21.8 | -12.9 | -17.7 | -12.1 | -10.6 | -17.3 | -5.3 | -8.3 |
| Lower 95% CI | -8.1 | -78.4 | -35.9 | -15.6 | -13.9 | -3.3 | -9.7 | -2.5 | -0.6 | -8.4 | 5.7 | 1.6 |
| Upper 95% CI | -25.7 | -81.5 | -46.7 | -29.5 | -28.4 | -20.8 | -24.4 | -20 | -18.8 | -24.6 | -14.2 | -16.4 |
| Female |  |  |  |  |  |  |  |  |  |  |  |  |
| Observed | 5366 | 1964 | 5231 | 6966 | 7322 | 7559 | 8589 | 8541 | 8676 | 8313 | 8858 | 9210 |
| Predicted | 6917 | 8910 | 8771 | 8512 | 8951 | 8050 | 9647 | 8879 | 8848 | 9029 | 8636 | 9156 |
| Change | -22.4 | -78 | -40.4 | -18.2 | -18.2 | -6.1 | -11 | -3.8 | -1.9 | -7.9 | 2.6 | 0.6 |
| Lower 95% CI | -13.7 | -76.1 | -34.7 | -9.9 | -10 | 4.9 | -2.1 | 7.1 | 9.6 | 3 | 15.7 | 13 |
| Upper 95% CI | -29.5 | -79.6 | -45.1 | -25.1 | -25 | -15 | -18.4 | -12.7 | -11.3 | -16.8 | -7.9 | -9.4 |
| 0-54 |  |  |  |  |  |  |  |  |  |  |  |  |
| Observed | 4892 | 1815 | 4634 | 6046 | 6216 | 6442 | 7285 | 7240 | 7382 | 6945 | 7507 | 7766 |
| Predicted | 6050 | 7916 | 7518 | 7330 | 7606 | 7005 | 8395 | 7729 | 7574 | 7759 | 7477 | 8093 |
| Change | -19.1 | -77.1 | -38.4 | -17.5 | -18.3 | -8 | -13.2 | -6.3 | -2.5 | -10.5 | 0.4 | -4 |
| Lower 95% CI | -9.4 | -75 | -32 | -8.5 | -9.4 | 3.3 | -4.2 | 4.7 | 9.5 | 0.6 | 13.7 | 7.8 |
| Upper 95% CI | -27 | -78.8 | -43.6 | -24.9 | -25.6 | -17.1 | -20.7 | -15.2 | -12.2 | -19.4 | -10.1 | -13.6 |
| 55-69 | 55-69 |  |  |  |  |  |  |  |  |  |  |  |
| Observed | 4295 | 1451 | 4012 | 5531 | 5798 | 6106 | 6769 | 6707 | 6739 | 6684 | 7193 | 7554 |
| Predicted | 5490 | 7290 | 6909 | 7151 | 7372 | 6787 | 7873 | 7207 | 7288 | 7667 | 7277 | 7814 |
| Change | -21.8 | -80.1 | -41.9 | -22.7 | -21.4 | -10 | -14 | -6.9 | -7.5 | -12.8 | -1.2 | -3.3 |
| Lower 95% CI | -13.5 | -78.5 | -36.5 | -15.6 | -14 | -0.5 | -6 | 3 | 2.5 | -3.6 | 10.3 | 7.3 |
| Upper 95% CI | -28.6 | -81.4 | -46.5 | -28.6 | -27.5 | -17.9 | -20.8 | -15.1 | -15.8 | -20.4 | -10.4 | -12 |
| 70- | 70- |  |  |  |  |  |  |  |  |  |  |  |
| Observed | 1102 | 331 | 1016 | 1477 | 1628 | 1665 | 1808 | 1720 | 1809 | 1810 | 1957 | 2117 |
| Predicted | 1352 | 1903 | 1957 | 1961 | 2058 | 1902 | 2214 | 2052 | 2102 | 2218 | 2116 | 2221 |
| Change | -18.5 | -82.6 | -48.1 | -24.7 | -20.9 | -12.5 | -18.3 | -16.2 | -13.9 | -18.4 | -7.5 | -4.7 |
| Lower 95% CI | -7.5 | -81 | -42.8 | -16.8 | -12.6 | -2.1 | -9.8 | -6.3 | -3.8 | -9 | 4 | 6.9 |
| Upper 95% CI | -27.1 | -84 | -52.5 | -31.2 | -27.8 | -20.8 | -25.4 | -24.1 | -22.2 | -26 | -16.8 | -14 |
| Operation |  |  |  |  |  |  |  |  |  |  |  |  |
| Observed | 1185 | 575 | 2055 | 2643 | 3010 | 3006 | 3268 | 3149 | 2699 | 2846 | 3070 | 2969 |
| Predicted | 1470 | 2759 | 2659 | 2635 | 2831 | 2422 | 2903 | 2578 | 2327 | 2706 | 2460 | 2435 |
| Change | -19.4 | -79.2 | -22.7 | 0.3 | 6.3 | 24.1 | 12.6 | 22.1 | 16 | 5.2 | 24.8 | 21.9 |
| Lower 95% CI | 3.2 | -76.4 | -10.6 | 16.6 | 23.2 | 48.7 | 31.3 | 46.3 | 43 | 26.3 | 53.8 | 51.5 |
| Upper 95% CI | -33.9 | -81.3 | -32 | -12 | -6.5 | 6.5 | -1.5 | 4.8 | -2.4 | -9.9 | 5 | 2 |
| Non-operation |  |  |  |  |  |  |  |  |  |  |  |  |
| Observed | 6667 | 2420 | 6117 | 8225 | 8181 | 8698 | 9849 | 10091 | 10516 | 10122 | 11914 | 13274 |
| Predicted | 8886 | 11401 | 10516 | 10481 | 10428 | 9927 | 11526 | 10920 | 11129 | 10816 | 10685 | 11060 |
| Change | -25 | -78.8 | -41.8 | -21.5 | -21.5 | -12.4 | -14.5 | -7.6 | -5.5 | -6.4 | 11.5 | 20 |
| Lower 95% CI | -13 | -76 | -33.1 | -9.1 | -8.5 | 3.8 | -0.8 | 8.9 | 11.6 | 11.7 | 34.1 | 44.1 |
| Upper 95% CI | -34 | -81 | -48.5 | -30.9 | -31.3 | -24.2 | -25 | -19.7 | -18 | -19.5 | -4.6 | 2.9 |
| Test |  |  |  |  |  |  |  |  |  |  |  |  |
| Observed | 3007 | 864 | 2462 | 3420 | 3660 | 3671 | 4042 | 3741 | 3854 | 3519 | 2693 | 2035 |
| Predicted | 3705 | 4617 | 4804 | 4833 | 5368 | 4691 | 5698 | 4931 | 4858 | 5631 | 5041 | 5848 |
| Change | -18.8 | -81.3 | -48.8 | -29.2 | -31.8 | -21.7 | -29.1 | -24.1 | -20.7 | -37.5 | -46.6 | -65.2 |
| Lower 95% CI | 4 | -76.8 | -36.6 | -12.3 | -17.4 | -2.1 | -15 | -6.1 | -1.4 | -24.8 | -34.1 | -58.4 |
| Upper 95% CI | -33.5 | -84.3 | -57 | -40.7 | -41.9 | -34.8 | -39.1 | -36.4 | -33.6 | -46.5 | -55.1 | -70.1 |

**Supplementary Table 3.** Differences in observed and predicted admissions after the COVID-19 lockdown according to cancer type

|  | January | February | March | April | May | June | July | August | September | October | November | December |
| --- | --- | --- | --- | --- | --- | --- | --- | --- | --- | --- | --- | --- |
| Breast |  |  |  |  |  |  |  |  |  |  |  |  |
| Observed | 1575 | 724 | 1623 | 1896 | 2037 | 2230 | 2489 | 2404 | 2714 | 2422 | 2568 | 2643 |
| Predicted | 2157 | 2207 | 2264 | 2033 | 2663 | 2496 | 2974 | 2757 | 2857 | 2720 | 2525 | 2653 |
| Change | -27 | -67.2 | -28.3 | -6.7 | -23.5 | -10.7 | -16.3 | -12.8 | -5 | -11 | 1.7 | -0.4 |
| Lower 95% CI | -14.9 | -61.7 | -15.2 | 14.1 | -10.2 | 7.2 | -1.9 | 4.5 | 13.9 | 8.7 | 27.5 | 24.3 |
| Upper 95% CI | -36.1 | -71.3 | -37.9 | -21.2 | -33.4 | -23.4 | -27 | -25.2 | -18.6 | -24.6 | -15.4 | -16.9 |
| Lung |  |  |  |  |  |  |  |  |  |  |  |  |
| Observed | 1882 | 543 | 1560 | 2154 | 2245 | 2446 | 2644 | 2518 | 2705 | 2530 | 2855 | 3024 |
| Predicted | 2358 | 2899 | 2815 | 2939 | 3123 | 2914 | 3383 | 3195 | 3210 | 3351 | 3290 | 3442 |
| Change | -20.2 | -81.3 | -44.6 | -26.7 | -28.1 | -16.1 | -21.8 | -21.2 | -15.7 | -24.5 | -13.2 | -12.1 |
| Lower 95% CI | -12 | -79.7 | -39.5 | -20.1 | -21.9 | -7.9 | -15.2 | -13.9 | -7.7 | -17.4 | -4.7 | -3.8 |
| Upper 95% CI | -27 | -82.6 | -48.8 | -32.3 | -33.4 | -22.9 | -27.5 | -27.4 | -22.5 | -30.4 | -20.3 | -19.2 |
| Stomach |  |  |  |  |  |  |  |  |  |  |  |  |
| Observed | 903 | 221 | 762 | 1158 | 1156 | 1153 | 1319 | 1188 | 1265 | 1180 | 1284 | 1403 |
| Predicted | 1300 | 1573 | 1562 | 1668 | 1707 | 1570 | 1791 | 1700 | 1732 | 1745 | 1684 | 1859 |
| Change | -30.5 | -86 | -51.2 | -30.6 | -32.3 | -26.6 | -26.4 | -30.1 | -27 | -32.4 | -23.8 | -24.5 |
| Lower 95% CI | -22.5 | -84.6 | -46.1 | -23.4 | -25.1 | -17.6 | -18.2 | -21.6 | -17.8 | -23.7 | -13.2 | -14.9 |
| Upper 95% CI | -37.1 | -87.1 | -55.5 | -36.5 | -38.2 | -33.8 | -33 | -37 | -34.2 | -39.3 | -32 | -32.2 |
| Lymphoid^a^ |  |  |  |  |  |  |  |  |  |  |  |  |
| Observed | 897 | 309 | 871 | 1231 | 1255 | 1272 | 1438 | 1397 | 1403 | 1379 | 1472 | 1651 |
| Predicted | 1148 | 1556 | 1517 | 1497 | 1530 | 1424 | 1663 | 1550 | 1632 | 1636 | 1558 | 1849 |
| Change | -21.9 | -80.1 | -42.6 | -17.8 | -18 | -10.7 | -13.5 | -9.9 | -14 | -15.7 | -5.5 | -10.7 |
| Lower 95% CI | -10.6 | -78.1 | -35.4 | -6.7 | -6.2 | 4.1 | -0.9 | 5.2 | 0.2 | -1.2 | 12.5 | 3.8 |
| Upper 95% CI | -30.6 | -81.9 | -48.3 | -26.5 | -27.1 | -21.8 | -23.3 | -21.2 | -24.7 | -26.5 | -18.6 | -21.6 |
| Gynecologic^b^ |  |  |  |  |  |  |  |  |  |  |  |  |
| Observed | 744 | 346 | 771 | 951 | 990 | 1038 | 1180 | 1151 | 1144 | 1127 | 1218 | 1248 |
| Predicted | 762 | 1173 | 1085 | 1142 | 1176 | 1020 | 1196 | 1111 | 1120 | 1196 | 1151 | 1147 |
| Change | -2.4 | -70.5 | -28.9 | -16.7 | -15.8 | 1.8 | -1.3 | 3.6 | 2.1 | -5.8 | 5.8 | 8.8 |
| Lower 95% CI | 17.8 | -66.8 | -18.5 | -5 | -3.9 | 19.2 | 13.2 | 20.6 | 19.2 | 9.1 | 23.7 | 27.7 |
| Upper 95% CI | -16.6 | -73.5 | -37 | -25.9 | -25.1 | -11.2 | -12.5 | -9.2 | -10.6 | -17.1 | -7.6 | -5.3 |
| Esophagus |  |  |  |  |  |  |  |  |  |  |  |  |
| Observed | 429 | 179 | 464 | 588 | 644 | 664 | 742 | 676 | 700 | 658 | 732 | 835 |
| Predicted | 477 | 935 | 770 | 806 | 864 | 793 | 982 | 849 | 804 | 924 | 788 | 916 |
| Change | -10.1 | -80.9 | -39.7 | -27 | -25.5 | -16.3 | -24.4 | -20.4 | -12.9 | -28.8 | -7.1 | -8.8 |
| Lower 95% CI | 21.7 | -77.9 | -27 | -12 | -10.7 | 2.8 | -10.7 | -2.5 | 8.5 | -13.5 | 17.9 | 12.1 |
| Upper 95% CI | -28.7 | -83.1 | -48.7 | -37.7 | -36.1 | -29.3 | -34.5 | -32.7 | -27.4 | -39.5 | -23.4 | -23.1 |
| Rectum |  |  |  |  |  |  |  |  |  |  |  |  |
| Observed | 555 | 180 | 519 | 723 | 735 | 737 | 787 | 759 | 736 | 746 | 782 | 811 |
| Predicted | 791 | 977 | 908 | 963 | 1028 | 913 | 1062 | 975 | 947 | 1002 | 1021 | 1043 |
| Change | -29.8 | -81.6 | -42.8 | -24.9 | -28.5 | -19.3 | -25.9 | -22.2 | -22.3 | -25.5 | -23.4 | -22.2 |
| Lower 95% CI | -21.6 | -79.8 | -36.3 | -16.5 | -20.5 | -8.5 | -17.1 | -11.5 | -10.8 | -14.8 | -12.2 | -10.8 |
| Upper 95% CI | -36.6 | -83 | -48.2 | -31.9 | -35.1 | -27.9 | -33 | -30.5 | -31.1 | -33.8 | -32 | -31 |
| Liver^c^ |  |  |  |  |  |  |  |  |  |  |  |  |
| Observed | 489 | 165 | 475 | 639 | 629 | 638 | 694 | 711 | 703 | 737 | 821 | 842 |
| Predicted | 575 | 764 | 680 | 714 | 751 | 686 | 750 | 713 | 728 | 766 | 765 | 799 |
| Change | -15 | -78.4 | -30.1 | -10.5 | -16.2 | -7 | -7.5 | -0.3 | -3.4 | -3.8 | 7.3 | 5.4 |
| Lower 95% CI | 1.3 | -75.4 | -18.7 | 3.4 | -3.9 | 8.3 | 6.5 | 15.7 | 11.7 | 10.6 | 23.5 | 20.7 |
| Upper 95% CI | -26.7 | -80.8 | -38.7 | -21.1 | -25.8 | -18.6 | -18.1 | -12.4 | -15 | -14.8 | -5.1 | -6.4 |
| Colon |  |  |  |  |  |  |  |  |  |  |  |  |
| Observed | 477 | 147 | 429 | 614 | 605 | 635 | 637 | 652 | 680 | 664 | 701 | 746 |
| Predicted | 695 | 763 | 796 | 794 | 841 | 765 | 836 | 788 | 816 | 818 | 830 | 851 |
| Change | -31.4 | -80.7 | -46.1 | -22.7 | -28.1 | -17 | -23.8 | -17.3 | -16.7 | -18.8 | -15.5 | -12.3 |
| Lower 95% CI | -21.7 | -78 | -37.5 | -8.8 | -14.7 | 1.8 | -7.1 | 3.5 | 4.7 | 3.2 | 8.2 | 12.6 |
| Upper 95% CI | -39 | -82.9 | -52.7 | -32.9 | -37.8 | -29.9 | -35.4 | -31.1 | -30.8 | -33.1 | -30.7 | -28.3 |

a: lymphoid, hematopoietic, and related tissue cancers; b: female gynecologic cancers; c: liver and intrahepatic bile ducts cancers
